# Aptamer-Functionalized Liposomes as a Potential Treatment for Basal Cell Carcinoma

**DOI:** 10.3390/polym11091515

**Published:** 2019-09-18

**Authors:** Anca N. Cadinoiu, Delia M. Rata, Leonard I. Atanase, Oana M. Daraba, Daniela Gherghel, Gabriela Vochita, Marcel Popa

**Affiliations:** 1 “Apollonia” University of Iasi, Faculty of Medical Dentistry, Department of Biomaterials, Pacurari Street, No. 11, Iasi 700511, Romania; jancaniculina@yahoo.com (A.N.C.); leonard.atanase@yahoo.com (L.I.A.); maria.mary2019@yahoo.com (O.M.D.); marpopa2001@yahoo.fr (M.P.); 2 NIRDBS - Institute of Biological Research Iasi, Department of Experimental and Applied Biology, Lascar Catargi 47, Iasi 700107, Romania; daniela_gherghel@yahoo.com (D.G.); gabriela.vochita@icbiasi.ro (G.V.); 3 Academy of Romanian Scientists, Splaiul Independentei Street, No. 54, Bucharest 050094, Romania

**Keywords:** liposomes, drug targeting, aptamer AS1411, basal cell carcinoma

## Abstract

More than one out of every three new cancers is a skin cancer, and the large majority are basal cell carcinomas (BCC). Targeted therapy targets the cancer’s specific genes, proteins, or tissue environment that contributes to cancer growth and survival and blocks the growth as well as the spread of cancer cells while limiting damage to healthy cells. Therefore, in the present study AS1411 aptamer-functionalized liposomes for the treatment of BCC were obtained and characterized. Aptamer conjugation increased liposome size, suggesting that the presence of an additional hydrophilic molecule on the liposomal surface increased the hydrodynamic diameter. As expected, the negatively charged DNA aptamer reduced the surface potential of the liposomes. Vertical Franz diffusion cells with artificial membranes were used to evaluate the in vitro release of 5-fluorouracil (5-FU). The aptamer moieties increase the stability of the liposomes and act as a supplementary steric barrier leading to a lower cumulative amount of the released 5-FU. The *in vitro* cell viability, targeting capability and apoptotic effects of liposomes on the human dermal fibroblasts and on the basal cell carcinoma TE 354.T cell lines were also evaluated. The results indicate that the functionalized liposomes are more efficient as nanocarriers than the non-functionalized ones.

## 1. Introduction

Cancer remains one of the world’s most devastating diseases, and skin cancer is the most common form of malignancy in the United States and many other countries [1,2]. There are three major types of skin cancer: melanoma, basal cell carcinoma (BCC), and squamous cell carcinoma (SCC). BCC, among one of the most common forms of human cancer, constitutes approximately 74% of skin cancer cases worldwide, and its incidence is increasing [3]. BCC is a malignant tumor localized in follicular germinative cells, which grows rapidly, causes destruction or damage of the surrounding tissue (in the area of the nose and ear) and can rarely cause metastasis to the lymph nodes, lung, bone, and liver [4]. Focused intervention, including cryotherapy, surgical excision, radiation therapy, photodynamic therapy, and chemotherapy (using antiproliferation drugs such as 5-aminolevulinic acid (5-ALA), 5-fluorouracil (5-FU), paclitaxel, resveratrol, imiquimod, temoporfin, and vismodegib) are usually used to combat the tumors [5,6,7,8,9]. Unfortunately, conventional chemotherapy applied to these conditions shows non-specific targeting, and high doses of anticancer drugs affect both normal and tumor cells, leading to the development of cellular resistance causing toxic effects (cytotoxicity, neurotoxicity, nephrotoxicity) throughout the entire body [10,11]. Over the past few decades, nanomedicine has had an enormous potential to improve the selectivity in targeting neoplastic cells by allowing the preferential delivery of drugs to tumors, and many type of nanoparticles have been studied for the treatment of skin cancers, including liposomes, dendrimers, polymersomes, chitosan-based nanocapsules, carbon-based nanoparticles, inorganic nanoparticles, and protein-based nanoparticles [1,12,13,14]. Liposomes are one of the most successful nanocarrier systems, which have been used for controlled and targeted drug delivery to cancer cells [15,16,17] and offer the potential to enhance the therapeutic index of anticancer agents, either by increasing the drug concentration in tumor cells and/or by decreasing the exposure in normal host tissues [18]. Liposomes are phospholipid vesicles that have a bilayered membrane structure similar to that of biological membranes, together with an internal aqueous core, and have been used to deliver a wide variety of small molecules, genes, imaging agents, and drugs [19,20,21,22,23]. Liposomes can be classified according to size and number of layers into multi-, oligo-, or unilamellar and show excellent circulation, penetration, and diffusion properties [1,24]. Various strategies have been adopted for targeting liposomes to the tumor sites, and active targeting via liposomal surface functionalization with ligands selective for receptors overexpressed in tumors is a good alternative to minimize anticancer agent toxicities to healthy cells [25]. Several types of cancer-targeting liposomes were prepared by functionalization with ligands that specifically interact with receptors highly expressed on cancer cells [23,26,27,28]. Liposomes functionalized with [D]-H6L9 and RGD (arginylglycylaspartic acid) peptides were obtained and tested for colon and breast cancer therapy [23]. AS1411 aptamer-functionalized liposomes increased cellular internalization and cytotoxicity to MCF-7 breast cancer cells and A375 malignant melanoma cells as compared to non-targeting liposomes [26,27,28]. A ligand used to modify liposomes should bind firmly to antigens that are selectively overexpressed on tumors, and these antigens should be present in sufficient quantities to induce an effect [29]. Surface functionalization of liposomes with aptamers (short ssDNA/RNA oligonucleotides having the ability to recognize target molecules by electrostatic interactions, hydrogen bonding, and hydrophobic interactions) has advantages over antibody grafting because the aptamers demonstrate higher target antigen recognition. Moreover, they are stable and smaller in size compared to antibodies, can be easily synthesized and chemically modified for molecular conjugation, and can be changed in sequence for improved selectivity [30,31].

The main purpose of this study was to develop a new type of AS1411 aptamer-functionalized liposomes loaded with 5-FU, which could present an excellent alternative for the treatment of BCC. The anticancer drug 5-FU blocks the methylation reaction of deoxyuridylic acid to thymidylic acid and is restricted by its systemic toxicities, severe gastrointestinal toxicities, hematologic side effects, and severe bone marrow disturbances [32]. AS1411 aptamer is a 26-mer DNA aptamer that specifically binds to nucleolin, a ~76 KDa protein highly expressed in the plasma membrane of different cancer cells, such as breast, melanoma, and prostate, but is not present in normal cells [33,34,35,36]. Our research group has already published a study in which the AS1411 aptamer-functionalized polymeric nanocapsules are obtained and characterized for BCC therapy [37]. The present work contains a very detailed study concerning the preparation of a series of liposomes loaded with 5-FU, drug release kinetics, evaluation of the in vitro cell viability, in vitro targeting capability and apoptotic effects of aptamer-functionalized liposomes on the human dermal fibroblasts cell line (HDF)and on the basal cell carcinoma TE 354.T cell line (BCC), used as model cells.

## 2. Materials and Methods 

### 2.1. Materials

Lipoid E PC S (Egg Yolk Phosphatidylcholine content: ≥96%) (PC) was received as a gift sample from Phospholipid GmbH (Köln, Germany); Cholesterol (CHOL), tris(2-carboxyethyl)phosphine hydrochloride (TCEP•HCl), 5-fluorouracil (5-FU), fluorescein disodium salt and Triton X were purchased from Alfa Aeser (Kandel, Germany); DSPE-PEG-Maleimide (DSPE-PEG-MAL) from Iris Biotech GmbH (Marktredwitz, Germany); DNA aptamer (AS1411-SH) was purchased from Integrated DNA Technologies, Inc. (Leuven, Belgium); chloroform was obtained from VWR Chemicals (Fontenay-sous-Bois, France). Human dermal fibroblasts cell line (HDFa) and the necessary supplies for in vitro cytotoxicity assay were purchased from Thermo Fisher Scientific (Bleiswijk, Netherlands).

### 2.2. Preparation Methods

#### 2.2.1. Preparation of Liposomes

The liposomes were prepared by film hydration method followed by sequential extrusion. Briefly, PC, CHOL, and DSPE-PEG-MAL, in different molar ratio (Table 1), were dissolved in chloroform in a round-bottom flask (250 mL). After dissolution, the solvent was evaporated under reduced pressure, at room temperature, in a rotary evaporator—Hei-VAP ML from Heidolph (Schwabach, Germany)—at 160 rpm, leading to the formation of a thin and homogeneous film of lipids on the surface of the flask.

The film obtained was then hydrated using 5 mL of 5-FU aqueous solution in phosphate-buffered saline (pH = 7.4) (10 or 15 mg/mL) at room temperature. After the vortex shaking, a spontaneous formation of multilamellar vesicles occurred. The dispersion was placed in a bath sonicator for 30 min at 25 °C in order to break the existing aggregates. Finally, the size of liposomes was reduced by multiple extrusion steps (10 times) through polycarbonate membranes with a pore size of 0.2 μm using a mini-extruder set from Avanti Polar Lipids, Inc (Alabaster, AL, USA). Free 5-FU was removed by dialysis method using a cellulose tubular membrane (12000–14000 Da). 

#### 2.2.2. Conjugation of AS1411 to the Surface of Obtained Liposomes

The selected aptamer AS1411 is promising for cancer therapy as it can target various pathways of nucleolin [33,34,35,36]. AS1411-SH was conjugated to the surface of liposomes using a method adapted from Li et al. [27]. The obtained liposomes with MAL-PEG-DSPE on the surface were incubated with AS1411-SH at a ratio of 5:1 for 24 h. The reaction was conducted in the presence of TCEP•HCl in the dark at room temperature with moderate stirring, under argon atmosphere. The conjugation efficiency between AS1411 and liposomes was determined by collecting non-conjugated aptamer after dialysis and measuring the concentration using a NanoDropOne UV–Vis Spectrophotometer from Thermo Fisher Scientific Inc (Madison, WI, USA).

### 2.3. Characterization 

Average hydrodynamic particle size(diameter) and polydispersity index (PDI) of the obtained liposomes were determined by dynamic light scattering (DLS) (Zetasizer Nano ZS from Malvern Panalytical, Worcestershire, UK) at 25 °C at a concentration of 1% (*w/v*), according to the standard ISO 13321: 1996. Five successive measurements were performed, and standard deviation was calculated. The zeta potential was measured by laser doppler micro-electrophoresis at 25 °C after dilution in the hydration medium to an appropriate counting rate. Transmission electron microscopy (TEM, PHILIPS CM100, Amsterdam, Netherlands) operating at an accelerating voltage of 100 kV was used to characterize the microstructure of the obtained liposomal vesicles. TEM samples were prepared by placing a drop of diluted dispersion of liposomes on a TEM grid and leaving them for air drying. The 5-FU encapsulation efficiency was spectrophotometrically (NanoDrop One Spectrophotometer from Thermo Scientific) determined after the destruction of liposomes in the presence of Triton X-100. Encapsulation efficiency was calculated using the following equation:

(1) $$\mathrm{Encapsulation}\;\mathrm{efficiency}\;\left(\%\right)=\frac{{\left(\mathrm{Drug}\;\mathrm{amount}\right)}_{\mathrm{final}}}{{\left(\mathrm{Drug}\;\mathrm{amount}\right)}_{\mathrm{initial}}}*\;100$$

In vitro drug release from liposomes was measured using Franz diffusion cells. A cellulose membrane (12,000–14,000 Da) was mounted between the donor and receptor compartments. The donor medium consisted of 0.5 mL of a liposomal formulation. The receptor medium consisted of 5 mL of saline phosphate buffer (pH = 7.4). The diffusion area between the donor and receptor compartments was 1.766 cm^2^. The stirring rate and temperature were maintained at 600 rpm and 32 °C, respectively. The obtained aptamer-functionalized liposomes were loaded into polymeric gels in order to study their efficiency as topical formulation, and therefore, these experiments were performed at 32 °C as it was demonstrated in the literature that this is the skin surface temperature [37,38], and the drug release was monitored over a time frame of 24 h. At appropriate intervals, 1 mL aliquots of the receptor medium were withdrawn and immediately replaced with an equal volume of fresh buffer. The amount of released drug was evaluated by UV–Vis spectroscopy (NanoDrop One Spectrophotometer from Thermo Scientific). The cumulative amount that permeated through the model membrane per unit area was calculated from the concentration of drug in the receiving medium and plotted as a function of time. The values are expressed as mean ± SE of three parallel measurements. For statistical analysis, the statistical significance of the cumulative amount that permeated through the model membrane per unit area and released percentage of 5-FU was analyzed by the ANOVA (analysis of variance). 

In vitro evaluation of aptamer-functionalized liposomes biocompatibility with blood components was performed using a method adapted from Vuddanda et al. [39]. The experimental procedure employed was approved by the Committee on Ethics of the “Apollonia” University of Iasi, Romania. The human blood sample used was freshly obtained from one healthy nonsmoker volunteer after institutional ethical clearance and appropriate informed consent. First, 5 mL of blood was centrifuged at 2000 rpm for 5 min. Supernatant plasma surface layer was removed, and the red blood cells (RBC) were separated and washed several times with normal saline solution. Then, the purified RBC were resuspended in normal saline solution to obtain 25 mL of RBC suspension. Then,2 mL of liposomes suspension in normal saline solution at different concentrations was added to 2 mL of RBC suspension (final concentrations were 100 μg lipids/mL, 250 μg lipids/mL and 500 μg lipids/mL). Positive (100% lysis) and negative (0% lysis) control samples were prepared by adding equal volumes of Triton X-100 2% and normal saline solution, respectively, to RBC suspension. The samples were incubated at 37 °C for 90, 180, and 300 min. The samples were slightly shaken once every 30 min to resuspend the RBC and liposomes. After the incubation time, the samples were centrifuged at 2000 rpm for 5 min, and 1.5 mL of supernatant was incubated for 30 min at room temperature to allow hemoglobin oxidation [40]. Oxyhemoglobin absorbance in supernatants was measured spectrophotometrically (Nanodrop One UV–Vis Spectrophotometer) at 540 nm. Hemolysis percentages of the RBC were calculated using the following formula:

(2) $$Haemolysis\;\left(\%\right)=\frac{\left(Abs_{sample}-Abs_{negative\;control}\right)}{\left(Abs_{positive\;control}-Abs_{negative\;control}\right)}$$

The experiments were performed in triplicate.

The in vitro cytotoxic effects of the obtained liposomes were determined on the human dermal fibroblasts cell line, according to procedure 10993-5:2009 [41] of the International Organization for Standardization (ISO). Briefly, HDF cells were cultured in Dulbecco’s modified Eagle’s Medium (DMEM), supplemented with 10% FBS, an antibiotic cocktail consisting of 1% (*v/v*) penicillin-streptomycin and 1% (*v/v*) nonessential amino acids. The medium was changed every 24 h. Cell incubation was carried out at 37 °C in a humidified atmosphere of 5% CO_2_ in air (incubator (air/CO_2_), MCO-5AC, Sanyo, Osaka, Japan). Cells were allowed to grow in 2 culture flasks (75 cm^2^) to 80% confluence and then were trypsinized with 0.25% trypsin solution at 37 °C for 3 min, followed by the addition of fresh medium at room temperature to neutralize trypsin. After centrifugation (centrifuge ROTOFIX-32A, Hettich, Beverly, MA, USA) and re-suspension in fresh medium, the viable cells were plated in flasks (25 cm^2^) and allowed to settle and attach for 24 h prior to treatment. After 24 h, aptamer-functionalized liposomes formulations (loaded with 5-FU and unloaded) at different lipids concentrations/mL fresh medium (100 μg; 250 μg, and 500 μg) were added. Fibroblasts viability after 72 h of incubation in culture media conditioned with the tested liposomes was determined. Cell viability was determined using the 0.4% trypan blue exclusion test and EVETM automatic cell counter. All procedures were performed in laminar flow hood (LAMIL PLUS 13, Kartusalan Metally Oy, Karstula, Finland) using sterile instruments, in triplicates. 

#### 2.3.1. Apoptosis Assay 

Normal human dermal fibroblasts (HDFa) were grown in Dulbecco’s modified Eagle’s Medium (DMEM) supplemented with 10% fetal bovine serum (FBS) and antibiotics (streptomycin/penicillin). They were kept at 37 °C, 5% CO_2_ in humidified air in an incubator. For the apoptosis tests, the cells were seeded into 12-well plates at a density of 50,000 cells/well and labeled using annexin V-FITC (fluorescein isothiocyanate) assay. Before the treatment with liposomes, for establishing the adherent monolayer confluence, HDF cell line was plated into 12-well plates for 24 h.

The apoptosis was investigated at 8 h after the treatment with aptamer-functionalized liposomes by annexin V-FITC/propidium iodide assay. Investigation of apoptosis by annexin V/propidium iodide resides in the strong affinity of annexin V for phosphatidylserine residues (normally hidden within the plasma membrane) on the surface of the cell. During apoptosis, phosphatidylserine is translocated from the cytoplasmic face of the plasma membrane to the cell surface. Propidium iodide is used to discriminate between dead and alive cells, also allowing separation between preapoptotic and apoptotic cells in combination with annexin V. Briefly, cells were detached by trypsinization, washed with cold PBS, resuspended in binding buffer and successively marked with annexin V-FITC and propidium iodide (provided with eBioscience kit).

The analysis of apoptosis was performed with a Beckman Coulter Cell Lab QuantaSC flow cytometer (Atlanta, GA, USA), equipped with a 488 nm laser, and the fluorescence was collected for FITC on FL1 (525 nm bandpass filter) and for propidium iodide on FL3 (670 nm long pass filter). All data were exported as LMD files and analyzed by Flowing Software v2.5.1 (Turku Center for Biotechnology, Turku, Finland) [42,43].

#### 2.3.2. Uptake of the Tested Liposomes by Shift in Fluorescence Intensity

For evaluation of cellular uptake of aptamer-functionalized liposomes, cell cultures (HDFa) were incubated 8 h in the presence of the fluorescein-labeled aptamer-functionalized liposomes, and the fluorescence intensity after incubation was measured by flow cytometry.

The fluorescein-labeled aptamer-functionalized liposomes were obtained using a fluorescein disodium salt (Flu) solution (4 mg/mL) in PBS for hydration of the lipid film at room temperature. All the steps to obtain the liposomes functionalized with aptamer are those already mentioned above in the preparation methods. Free Flu was removed by dialysis method using a cellulose tubular membrane (12000–14000 Da). The washing water was changed until the free fluorescein was no longer detected spectrophotometrically. Immediately after the washing steps was evaluated the cellular uptake to avoid the release of fluorescein. The Flu encapsulation efficiency was spectrophotometrically evaluated. 

#### 2.3.3. In Vitro Antitumoral Effect on Basal Cell Carcinoma 

The human basal cell carcinoma cell line TE 354.T (ATCC® CRL-7762™)was cultivated in Dulbecco’s Modified Growth Medium (DMEM, Biochrom AG, Berlin, Germany), supplemented with 10% fetal bovine serum (FBS, Sigma, Taufkirchen, Germany), 100 μg/mLstreptomycin (Biochrom AG, Berlin, Germany), 100 IU/mL penicillin Biochrom AG, Germany) in a humidified atmosphere of 5% CO_2_ at 37 °C.Cell viability of the BCC cells incubated with obtained liposomes was determined by MTT method, according to Mosmann [44] and Laville et al. [45]. This test is based on the ability of mitochondrial dehydrogenases of living cells to convert the water-soluble yellow substrate (MTT) to dark blue. Moreover, the amount of insoluble formazan is directly proportional to the number of live cells [46]. The cells were trypsinized according to standard trypsinization procedure with trypsin/EDTA, then counted and resuspended in 96-well microplates (7 × 10^3^ cells/well), in the same temperature and humidity conditions. After monolayer formation (24 h), the cells were treated for 48 h with the unloaded and loaded with 5-FU liposomes in different doses (25; 50; 75; 100 μg/mL). After treatment, the samples were processed by MTT assay, the absorbance being measured at 570 nm using the Biochrom EZ Read 400 microplate automatic reader (Cambridge, UK). The cell viability percentage was calculated according to the equation:

Cell viability (%) = Absorbance(Test)/Absorbance(Control)*100(3)

## 3. Results and Discussion

**Figure 1:**
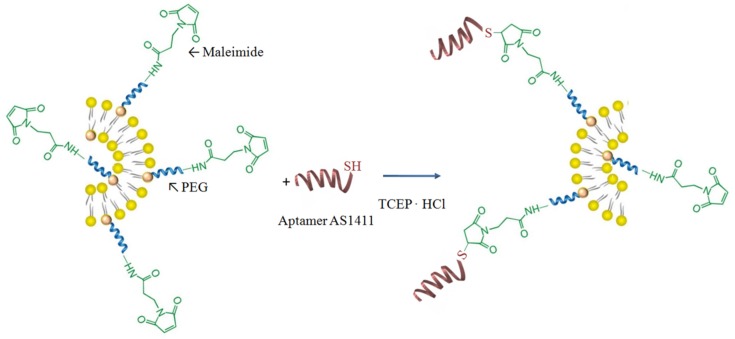
Schematic illustration of the AS1411 aptamer-functionalized liposomes.

Liposomes composed of PC, Chol, and DSPE-PEG-MAL were prepared, and then aptamer AS1411, which specifically targets tumor cells, was conjugated onto the external surface of liposomes. Aptamers are capable of binding to a variety of molecular targets with high affinity and specificity; therefore, the conjugation of aptamers to liposomes enhances the active targeting and thus the personalized medicine in order to reduce the effects on non-target tissues. Figure 1 illustrates the basic design and formulation of AS1411-SH/liposomes conjugates. 

Maleimides from the surface of the liposomes are electrophilic compounds that show high selectivity towards thiols. However, thiols are prone to oxidative dimerization with the formation of disulfide bonds, which prevent the reaction with maleimides. Therefore, it is necessary to reduce disulfides prior to the conjugation and to exclude oxygen from the reaction. TCEP (tris-carboxyethylphosphine) reagent was used in order to reduce the disulfides bonds. As a function of the molar ratios between PC, CHOL, and DSPE-PEG-MAL, different aptamer-functionalized liposome samples were prepared, and the conjugation efficiency values, determined by UV–Vis spectroscopy, are provided in Table 2.

From Table 2 it appears that the highest conjugation efficiency was obtained for the liposomes with the highest amount of DSPE-PEG-MAL. On the contrary, it seems that a small amount of PC and CHOL, which is used to increase the hydrophobic-hydrophobic interactions in lipids bilayer, leads to lower conjugation efficiency. Moreover, it can be observed that the variation of the encapsulated drug amount, from 10 to 15 mg/mL, has only a slight effect on this conjugation efficiency. 

### 3.1. Size and Zeta Potential

DLS was used to evaluate the sizes and surface zeta potentials of the obtained liposomes. Table 3 presents the size, polydispersity index (PDI), and zeta potential values of non- and AS1411 aptamer-functionalized liposomes. 

As a general remark, it appears from Table 3 that the drug-loaded non-functionalized liposomes have an average hydrodynamic diameter of 176 ± 20 nm and a relatively low polydispersity (around 0.1). It also seems that the ratio between PC/Chol/DSPE-PEG-MAL has no direct influence on the liposome’s size. Moreover, the conjugation of the AS1411 aptamer on the surface of drug-loaded liposomes leads to an increase of the liposome’s size, to around 190 ± 15 nm, as it will be expected in the presence of an additional hydrophilic molecule [40]. Furthermore, the aptamer increased the surface potential of the liposomes, indicating the attachment of negatively charged DNA strands on the liposome’s surface [26]. This increase of the absolute ZP values provides a higher stability. These results are in good correlation with those obtained by different authors [26,27,47]. Finally, from Table 3 it can be observed that increasing the amount of loaded drug, from 10 to 15 mg/mL, has only a slight consequence on the liposome’s size. A typical TEM photo of the drug-loaded liposomes is provided in Figure S1 (Supplementary Materials).

### 3.2. 5-FU Encapsulation Efficiency

Liposome suspension (20 μL) was diluted with 4 mL PBS (pH = 7.4), and then 500 μL of 1% Triton X-100 solution was added in order to destroy the liposomes and to release the 5-FU. The amount of 5-FU was evaluated spectrophotometrically (λ = 266 nm) (NanoDrop One, Thermo Scientific) by taking into account the equation of the standard curve. The drug encapsulation efficiency was calculated taking into account the initial amount of drug from the hydration solution. Table 4 lists the encapsulation efficiency of 5-FU in the non- and AS1411-functionalized liposomes prepared at different drug/lipid ratios. 

The results provided in Table 4 show that the encapsulation efficiency remains almost constant, within the experimental error limits, after the functionalization step of the liposomes with the aptamer. 

### 3.3. In Vitro Drug Release 

**Figure 2:**
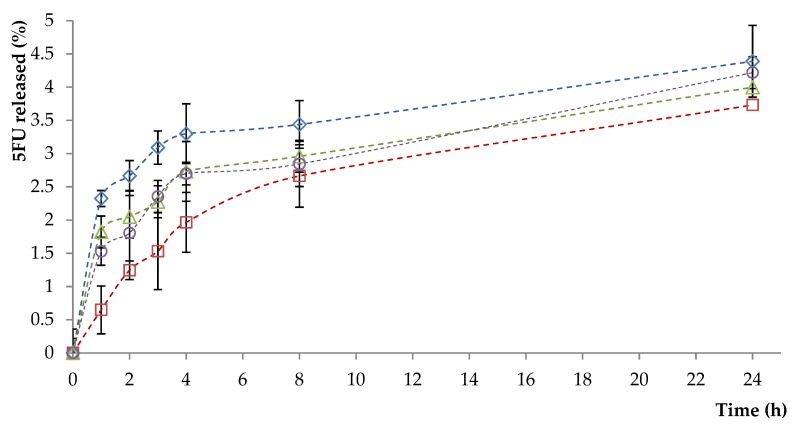
The release profiles (%) of 5-FU from the non-functionalized liposomes. Diamonds—L1-5FU-10; squares—L3-5FU-10; triangles—L3-5FU-15, circles—L4-5FU-15.

The release characteristics of 5-FU, as a function of time, from non- and AS1411 aptamer-functionalized liposomes were studied spectrophotometrically. Figure 2 shows the release profiles (%) of 5-FU from the non-functionalized liposomes that were prepared at different drug/lipid ratios. From this figure it can be observed that the sample L3-5FU-15 released a higher percentage of 5-FU than sample L3-5FU-10, which is correlated with the amount of drug encapsulated in liposomes. Samples L3-5FU-15 and L4-5FU-15 have a similar release profile, which indicates that lipids liposome composition does not influence the drug release profile at identical initial loaded drug amount.

**Figure 3:**
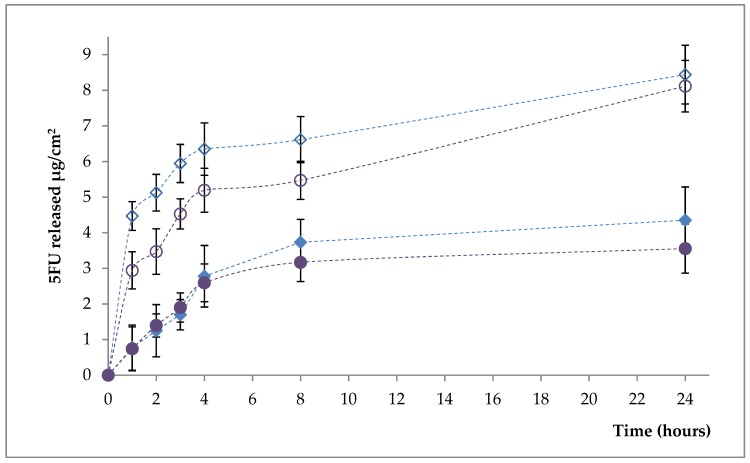
The cumulative amount that permeated through the model membrane per unit area (L1-5FU-10—open diamond; L4-5FU-15—open circle; L1Apt-5FU-10—full diamond; L4Apt-5FU-15—full circle).

Functionalization of liposomes with AS1411-SH aptamer increases their stability, as evidenced by a decrease in the percentage of drug released from functionalized liposomes after 24 h (Figure S2, Supplementary Materials). The cumulative amount that permeated through the model membrane per unit area was also calculated from the concentration of drug in the receiving medium and plotted as a function of time (Figure 3 and Figure S3).

From Figure 3 and Figure S3 (Supplementary Materials) it appears that the release amount of 5-FU is lower, at equal times, for the liposome samples functionalized with the aptamer than for the non-functionalized samples. A possible explanation of this behavior is based on the fact that the aptamer moieties increase the stability of the liposomes and also act as a supplementary steric barrier.

Statistical results provided in Tables S1 and S2 (Supplementary Materials) for the two analyzed samples shows that the relationship between the cumulative mass of 5-FU released and time is valid. 

### 3.4. In Vitro Evaluation of Liposomes Biocompatibility with Blood Components 

**Figure 4:**
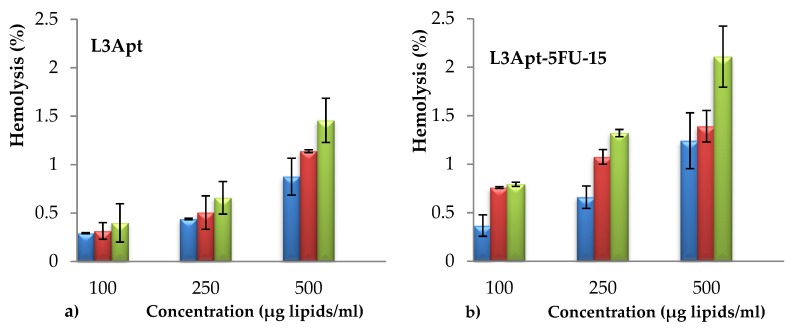
Hemolysis percentage after 90 (blue bar), 150 (red bar), and 300 min (green bar) exposure to L3Apt (**a**) and L3Apt-5FU-15 (**b**) liposomes sample.

Determination of hemolytic properties is one of the most common tests in studies of liposomes’ interaction with blood component. The obtained AS1411liposomes (with and without encapsulated drug) were incubated in blood at different concentrations. The hemoglobin released by damaged cells was measured at three different time intervals (90 min, 180 min, and 300 min). The results of hemolysis assay are shown in Figure 4. The results were expressed as means ± SD (*n* = 3).

From Figure 4 it can be observed that the hemolytic percentage increases with the increasing concentration of liposomes. A sample is considered as hemolytic if the hemolytic percentage is above 5%. It clearly appears from Figure 4 that the hemolytic percentage was lower than 5% for all tested concentrations, at all three tested times. From these tests it can be concluded that the prepared liposomes are hemocompatible. 

### 3.5. In Vitro Cytotoxic Effects

**Figure 5:**
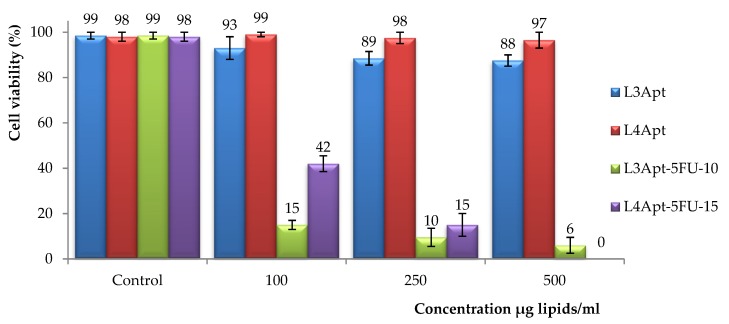
Viability of fibroblast cells after 72 h of incubation in culture media with L3Apt (with and without drug included) and L4Apt (with and without drug included).

For in vitro cytotoxicity assessment of aptamer-functionalized liposomes (with and without encapsulated drug), human fibroblast cells (HDFa) were used as model cells. The effects of liposomes on viability of fibroblasts after an incubation period of 72 h are shown in Figure 5.

Cytotoxicity has shown a concentration-dependent effect on tested cells, and concentration increase leads to a decrease of the viability. A higher amount of DSPE-PEG-MAL lipid in the initial mixture results in an increase in cell viability (Figure 5). As expected, a strong inhibition of cell proliferation was observed in the case of functionalized liposomes loaded with 5-FU, an effect which was induced by the presence of 5-FU.

**Figure 6:**
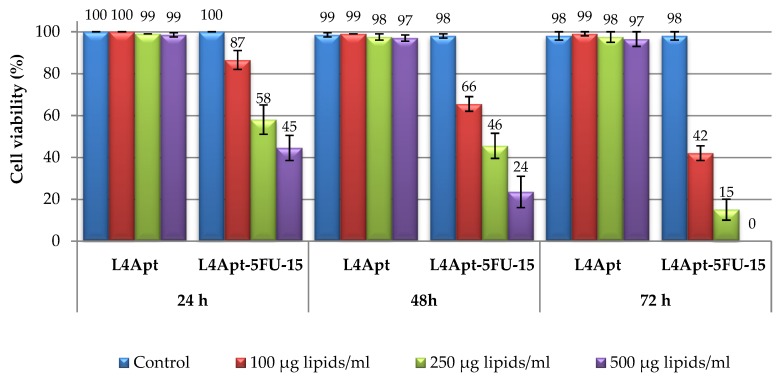
Viability of fibroblast cells after 24, 48, and 72 h of incubation in culture media with L4Apt (with and without drug included).

After analyzing the obtained results, it was decided to use the L4Apt-5FU-15 sample for the following tests because it showed a better cell viability, compared with the L3Apt-5FU-10 sample, and our objective is the destruction of the cancer cells and also the protection of the normal cells. Figure 6 shows the effects of L4Apt sample (with and without drug included) on viability of HDFa after incubation periods of 24, 48, and 72 h. The sample L4Apt shows good compatibility with the human fibroblast cells (cell viability being over 97%) at all concentrations and incubation times tested. In the case of sample L4Apt-5FU-15, it can be observed that cell viability decreases with increasing lipid concentration, as expected, because with increasing lipid concentration the amount of 5-FU increases. Cell viability also decreases with increasing incubation time, and this can be explained by the release of the drug from liposomes.

### 3.6. Apoptosis Evaluation

**Figure 7:**
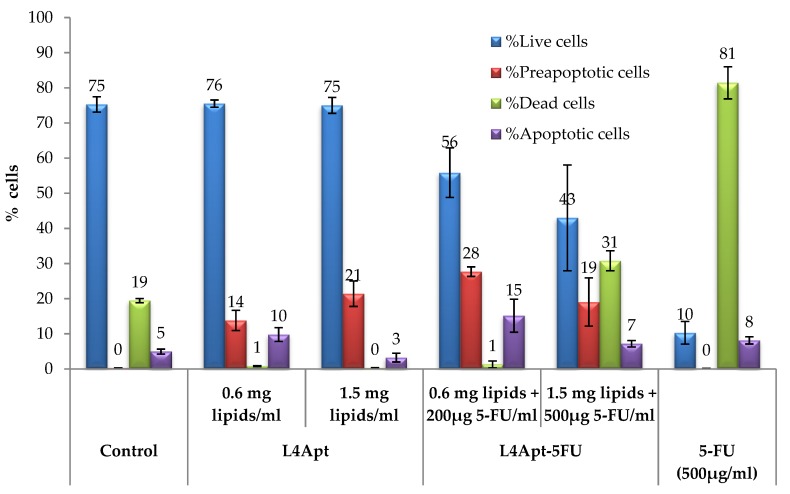
Percentage distribution of the viable, dead, apoptotic, and preapoptotic cells at 8 h after the treatment with the aptamer-functionalized liposomes loaded or not loaded with 5-FUas quantified by annexin V-FITC and propidium iodide in apoptosis assay (flow cytometric method) according to every experimental treatment.

Incubation of the normal human dermal fibroblasts (HDFa) with different concentrations of aptamer-functionalized liposomes (L4Apt) or 5-FU-loaded aptamer-functionalized liposomes (L4Apt-5FU-15) has led to varying degrees of apoptosis (Figure 7).

From Figure 7 it can be noted that simple liposomes caused an increase in preapoptotic cell frequency compared to the control. Loading the liposomes with 5-FU has resulted in a moderate increase in preapoptotic cell frequency, similar to that registered in the case of liposomes. Apoptotic cell frequencies show positive variations in L4Apt-5FU-15-treated cells, resulting in a significant increase compared to the control alone.

Significant increases were seen in the frequency of dead cells in the case of L4Apt-5FU-15 (1.5 mg lipids + 500 μg 5-FU/mL), which far exceeded the threshold set for the witness. 

Compared to the 5-FU effect on cell cultures, its inclusion in the liposomes leads to a gradual release because in the same time range (8 h), 5-FU impact is rapid (81% dead cells), whereas inclusion in liposomes has led to the induction of the cell apoptosis process, as can be seen from the evolution of the preapoptotic and apoptotic cell frequency (Figure S5).The existence of a direct liposome effect should not be neglected, as can be seen in the case of L4Apt sample, where the effect of inducing apoptosis is consistent, preapoptotic cells frequency being higher than that registered in the case of control group.

### 3.7. Uptake of the Tested Liposomes by Shift in Fluorescence Intensity

**Figure 8:**
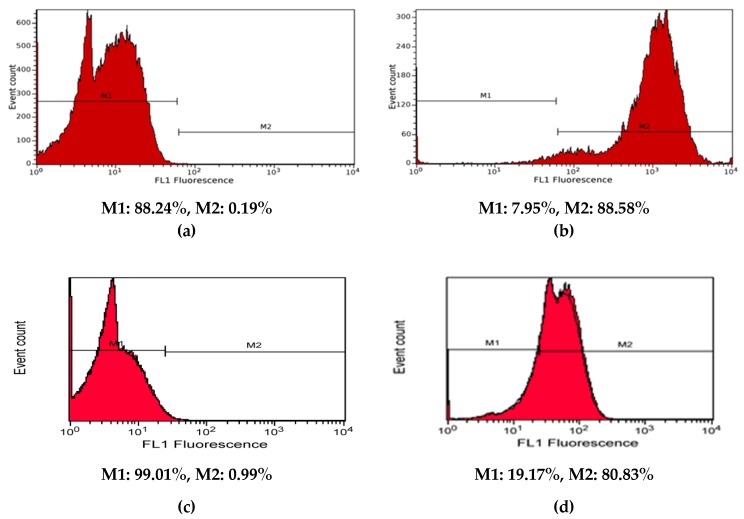
Histograms of control cells (**a and c**), of the cells treated with liposomes loaded with fluorescein (**b**) and of the cells treated with aptamer-functionalized liposomes loaded with fluorescein (**d**) in order to evaluate their uptake by human dermal fibroblast (HDF) cells. M1—negative for fluorescein, no uptake; M2—positive for fluorescein, cells are loaded with liposomes or aptamer-functionalized liposomes labeled with fluorescein.

To assess the targeting capability of aptamer-functionalized liposomes, fluorescein-loaded liposomes (L4Apt-Flu Sample) were incubated with normal human dermal fibroblasts (HDF), and the fluorescence of the treated cells was analyzed by flow cytometry (Figure 8). In order to compare their binding affinity with HDF cells, a low sample concentration (0.6 mg lipids/mL) and a time incubation of 8 h were used. 

Compared to the unlabeled control, fluorescein-labeled liposomes (L4Apt-Flu Sample) exhibit greater fluorescence intensity, as can be seen from the histograms (Figure S5), indicating their penetration into the cell. In the case of liposomes loaded with fluorescein and not functionalized with the aptamer, the uptake rate was increased, but always smaller than in the case of the functionalized liposomes. 

### 3.8. In Vitro Antitumoral Effect on Basal Cell Carcinoma 

**Figure 9:**
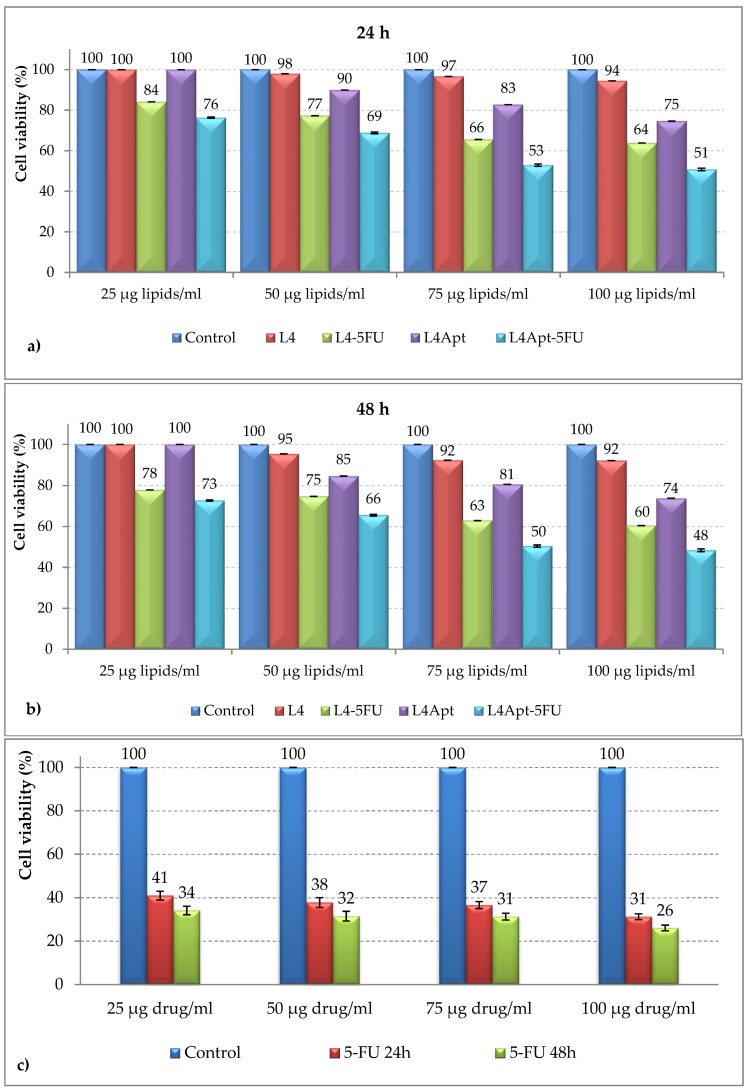
Cell viability of TE 354.T cells as incubated with L4, L4-5FU-15, L4Apt, L4Apt-5FU-15 in doses of 25, 50, 75, and 100 µg lipids/mL for 24 h (**a**) and 48 h (**b**)and 5-FU in doses of 25, 50, 75, and 100 µg drug/mL for 24 and 48 h (**c**) determined by MTT test.

The in vitro assessment of antineoplastic effect of aptamer-functionalized liposomes (loaded or not loaded with drug) was based on the use of basal cell carcinoma. The cells were incubated for 48 h in the presence of liposomes. The data are shown in Figure 9. 

The non-cytotoxic effect of L4 sample on TE 354.Tcell line, similar to the untreated control, is observed at both 24 and 48 h of treatment. In the case of aptamer-functionalized liposomes (L4-Apt), a slight increase in cytotoxicity is observed at the dose of 100 µg/mL and after 48 h of treatment. The drug alone demonstrates a pronounced cytotoxic effect on cancer cells even at low concentrations (25 μg/mL). Moreover, the free drug also destroys the healthy cells. The drug encapsulated in the liposomes is gradually released, overcoming the cytotoxicity of 5-FU alone, as can be seen in the case of the sample L4-5FU. The L4Apt-5FU-15induces a more pronounced cytotoxic effect on TE 354.T cells, compared to L4-5FU-15, thus demonstrating the efficiency of liposomes functionalization with AS1411aptamer.Liposomes that are functionalized with aptamer AS1411were also found to be an attractive therapeutic alternative for breast cancer [26,47] and melanoma [27].

## 4. Conclusions

Aptamer-functionalized liposomes were designed to improve therapeutic effects of 5-FU administration by eliminating a series of secondary, undesired effects, determined by the classical administration, and to improve health condition of people with BCC. AS1411 conjugation increased liposome size, suggesting that the presence of an additional hydrophilic molecule on the liposomal surface increased the hydrodynamic diameter. As expected, the negatively charged DNA aptamer increased the surface potential of the liposomes, leading to increased stability. The in vitro release kinetics of 5-FU have revealed that after 24 h, only a small amount of drug, between 3 and 5 µg/cm^2^, was released from the aptamer-functionalized liposomes. Cell viability study suggested that the drug-free liposomes have good biocompatibility. The higher fluorescence intensity of the HDF cells treated with fluorescein-labeled aptamer-functionalized liposomes has demonstrated that these liposomes are able to penetrate the cells. In vitro antitumoral tests indicate that the aptamer functionalized liposomes loaded with 5-FU induce a more pronounced cytotoxic effect on TE 354.T cells, compared to the non-functionalized ones, thus demonstrating the efficiency of liposomes functionalization with AS1411 aptamer. After a rigorous analysis of the obtained results, it was decided that the L4Apt-5FU-15 sample represents the optimal combination in terms of lipid composition and the lipids/drug ratio that can be used for the further trials tests. 

## Figures and Tables

**Table 1:** The experimental plan used for the preparation of liposome samples loaded with 5-fluorouracil (5-FU).

Sample	PC (mmol)	Chol (mmol)	DSPE-PEG-MAL (mmol)	PBS (mL)	5-FU in PBS (mg/mL)	AS1411-SH (mmol)
L1-5FU-10	15	9	1	5	10	-
L2-5FU-10	15	13.5	1	5	10	-
L3-5FU-10	10	6	1	5	10	-
L3-5FU-15	10	6	1	5	15	-
L4-5FU-10	10	6	1.5	5	10	-
L4-5FU-15	10	6	1.5	5	15	-
L1Apt-5FU-10	15	9	1	5	10	1
L2Apt-5FU-10	15	13.5	1	5	10	1
L3Apt-5FU-10	10	6	1	5	10	1
L4Apt-5FU-10	10	6	1.5	5	10	1.5
L4Apt-5FU-15	10	6	1.5	5	15	1.5

**Table 2:** The conjugation efficiency values for aptamer-functionalized liposome samples.

Sample	Conjugation Efficiency (%)
L1Apt-5FU-10	36.2 ± 3.5
L2Apt-5FU-10	37.3 ± 3.8
L3Apt-5FU-10	28.0 ± 3.0
L4Apt-5FU-10	39.6 ± 2.9
L4Apt-5FU-15	37.2 ± 3.7

**Table 3:** Size, polydispersity index (PDI), and zeta potential values of non- and AS1411 aptamer-functionalized liposomes. Data are presented as mean ± SD (*n* = 5 in each group).

Sample	Mean Diameter (d.nm)	PDI	Zeta Potential (mV)
L1	182 ± 10	0.12	−0.4 ± 0.1
L2	187 ± 22	0.18	−0.4 ± 0.5
L3	182 ± 12	0.15	−0.5 ± 0.2
L1-5FU-10	184 ± 12	0.10	−4.5 ± 0.3
L2-5FU-10	189 ± 23	0.18	−0.4 ± 0.9
L3-5FU-10	170 ± 11	0.08	−5.6 ± 0.7
L3-5FU-15	185 ± 12	0.15	−0.4 ± 0.1
L4-5FU-10	164 ± 33	0.11	−5.3 ± 0.3
L4-5FU-15	170 ± 18	0.14	−6.4 ± 0.9
L1Apt-5FU-10	200 ± 16	0.36	−9.6 ± 1.5
L2Apt-5FU-10	197 ± 15	0.13	−12.0 ± 2.4
L3Apt-5FU-10	190 ± 9	0.20	−11.1 ± 1.2
L4Apt-5FU-10	172 ± 21	0.09	−12.4 ± 1.6
L4Apt-5FU-15	182 ± 27	0.16	−12.9 ± 1.3

**Table 4:** The encapsulation efficiency of 5-FU in the non- and AS1411 functionalized liposomes.

Sample	Encapsulation Efficiency of 5-FU (%)
L1-5FU-10	7.8 ± 0.9
L3-5FU-10	7.5 ± 0.9
L3-5FU-15	8.5 ± 1.1
L4-5FU-15	8.7 ± 1.4
L1Apt-5FU-10	7.2 ± 0.2
L3Apt-5FU-10	7.1 ± 1.2
L3Apt-5FU-15	8.1 ± 0.8
L4Apt-5FU-15	8.3 ± 1.0

## Supplementary Materials

The following are available online at https://www.mdpi.com/2073-4360/11/9/1515/s1, Table S1: Summary of the output of the analysis of variance concerning the linear regression of the cumulative mass of 5-FU released and time for the samples L1-5FU-10 and L1Apt-5FU-10, Table S2:Summary of the output of the analysis of variance concerning the linear regression of the cumulative mass of 5-FU released and time for the samples L3-5FU-10 and L3Apt-5FU-10,Figure S1: TEM photograph of the liposomes, Figure S2: 5-FU release kinetics from non-functionalized liposomes, Figure S3: The cumulative amount that permeated through the model membrane per unit area after 7 days, Figure S4: Viability of fibroblast cells in the presence of L3 (with and without drug included) and L4 (with and without drug included), Figure S5:Analysis of apoptosis based on double stain of the cells with annexin V-FITC and propidium iodide and their corresponding dotplots
