# Supplementary material for: Aptamer-Functionalized Liposomes as a Potential Treatment for Basal Cell Carcinoma
Source: Polymers (Basel). 2019 Sep 18;11(9):1515. doi: 10.3390/polym11091515 (PMC6780726; doi:10.3390/polym11091515)
Supplement: Supplementary file 1 [file polymers-11-01515-s001.pdf]

Supplementary data
Statistical analysis

Table S1: Summary of the output of the analysis of variance concerning the linear regression of the cumulative mass of 5-FU released and time for the samples L1-5FU-10 and L1Apt-5FU-10 (level of significance = 0.05)

**Source**	**Sum of squares (SS)**	**df**	**Mean square (MS)**	**F**	**F critical**
**Between groups**	351.972	1	351.972	0.58919	4.25968
**Within groups**	14,337.2	24	597.385		
**Total**	14,689.2	25			

Table S2: Summary of the output of the analysis of variance concerning the linear regression of the cumulative mass of 5-FU released and time for the samples L3-5FU-10 and L3Apt-5FU-10 (level of significance = 0.05)

**Source**	**Sum of squares (SS)**	**df**	**Mean square (MS)**	**F**	**F critical**
Between groups	225.918	1	225.918	0.43106	4.25968
Within groups	12,578.3	24	524.094		
Total	12,804.2	25			

TEM photograph of the liposomes

The TEM photograph of the liposomes is presented in Figure S1, and it appears that the liposomes have a spherical shape with a single double-layered lipid membrane.

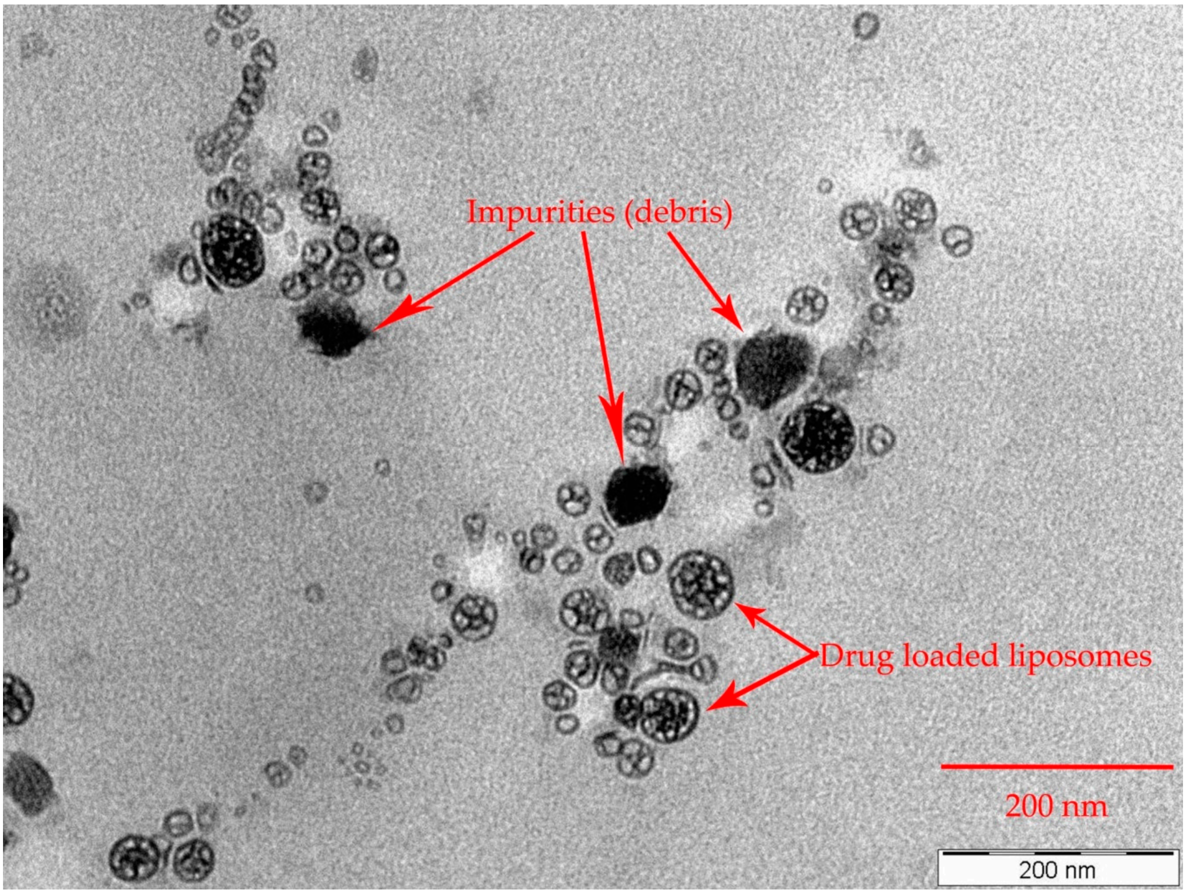

Figure S1. TEM photograph of the liposomes loaded with 5-FU (L2-5FU-10 sample)

5-FU release kinetics from non-functionalized liposomes

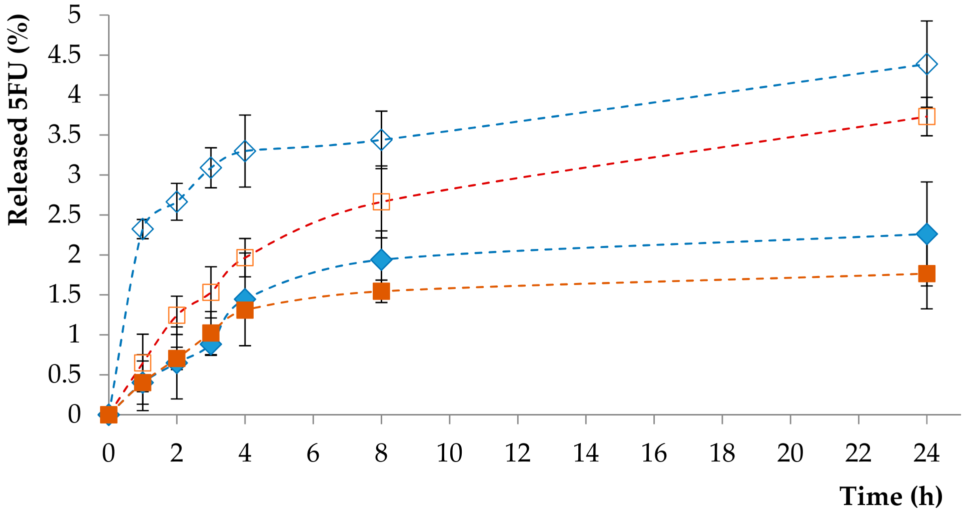

Figure S2. The 5-FU release kinetics from non-functionalized liposomes (L1-5FU-10—open diamond and L3-5FU-10—open squares) compared to aptamer-functionalized liposomes (L1Apt-5FU-10—full diamond and L3Apt-5FU-10—full squares)

The cumulative amount that permeated through the model membrane per unit area after 7 days

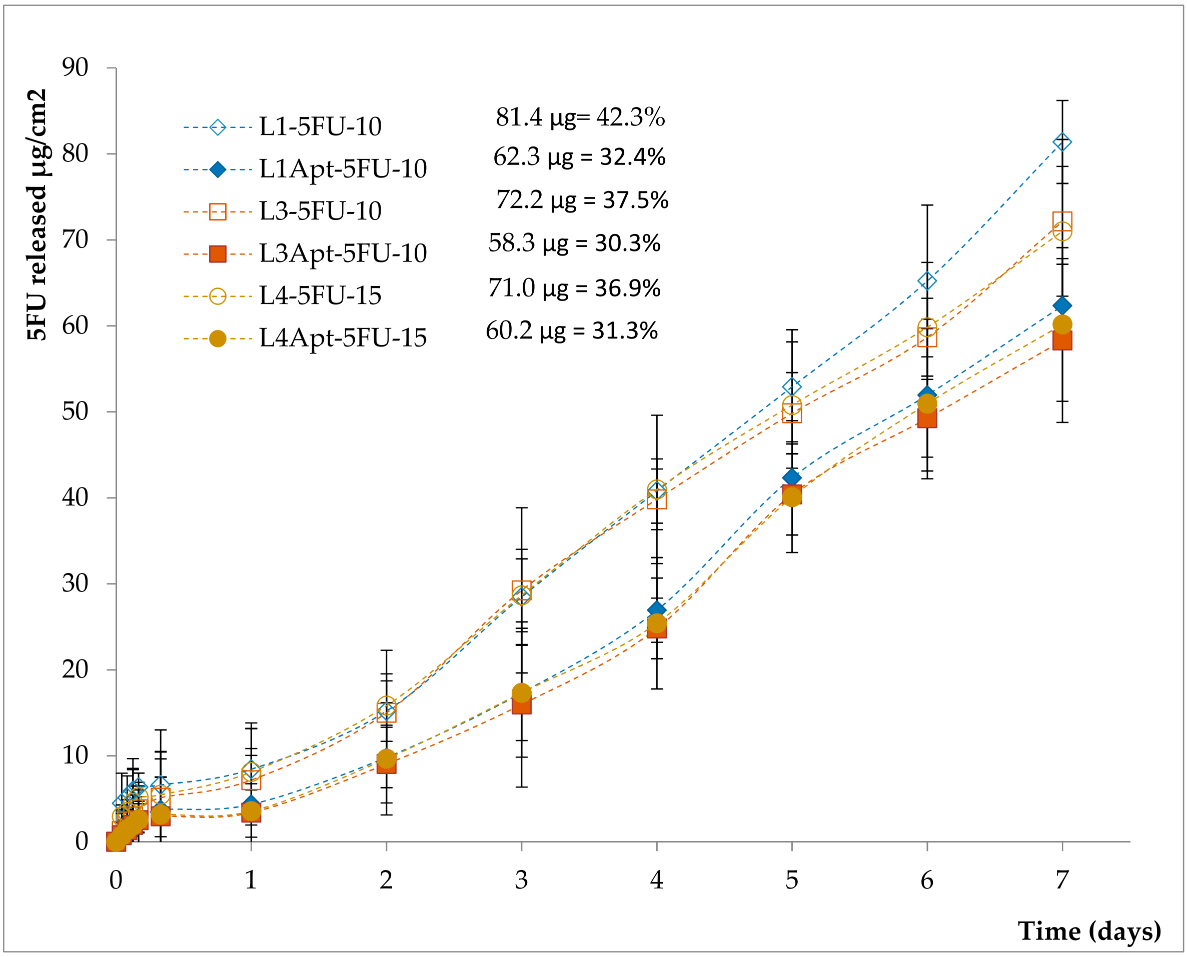

Figure S3. The cumulative amount that permeated through the model membrane per unit area after 7 days

In vitro cytotoxic effects

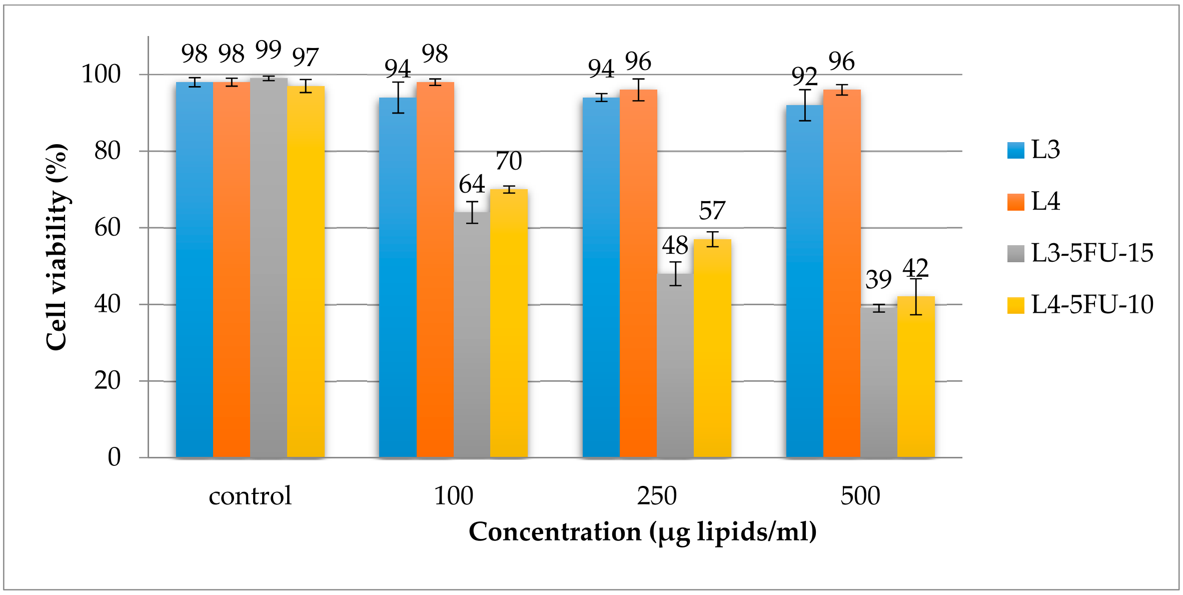

Figure S4. Viability of fibroblast cells in the presence of L3 (with and without drug included) and L4 (with and without drug included)

The analysis of apoptotis based on double stain of the cells with annexin V-FITC and propidium iodide and their corresponding dotplots.

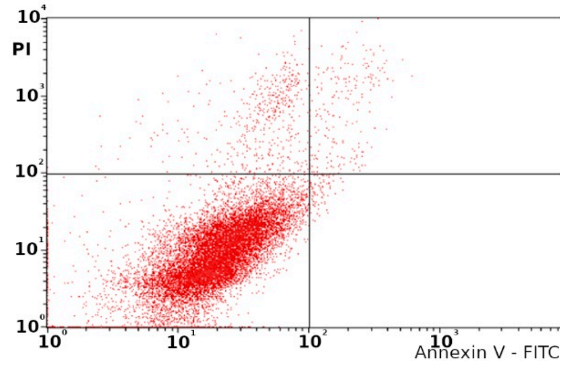

A - control group

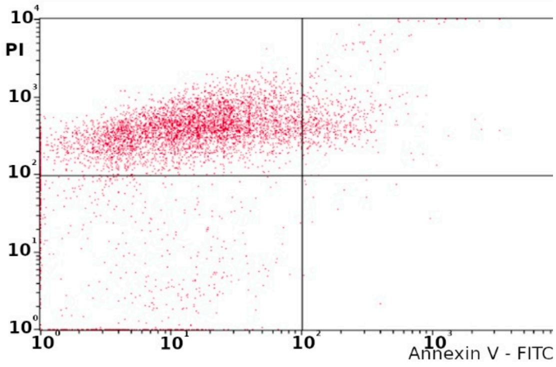

B - 5-FU treated groups

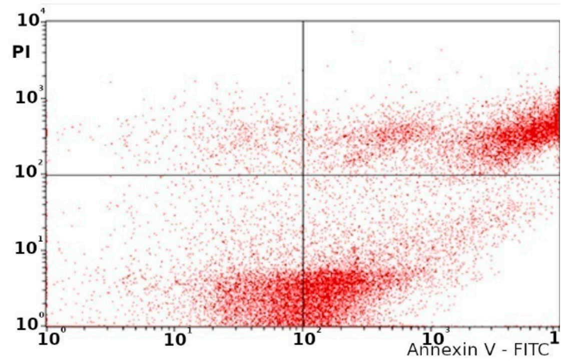

C - L4Apt (0.6 mg lipids/mL)

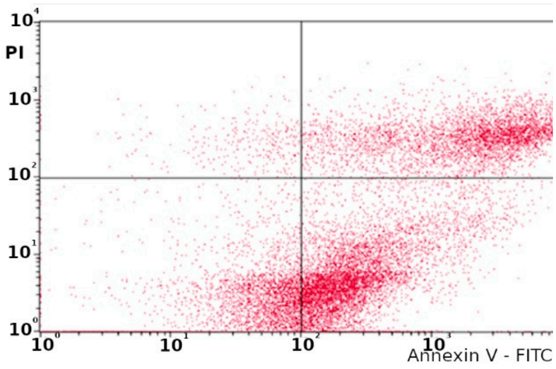

D - L4Apt (1.5 mg lipids/mL)

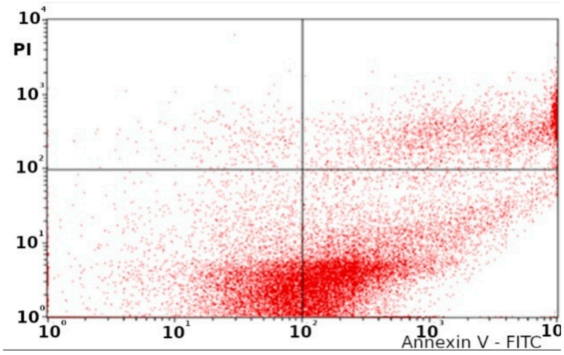

E - L4Apt-5FU(0.6 mg lipids + 200 µg 5-FU/mL)

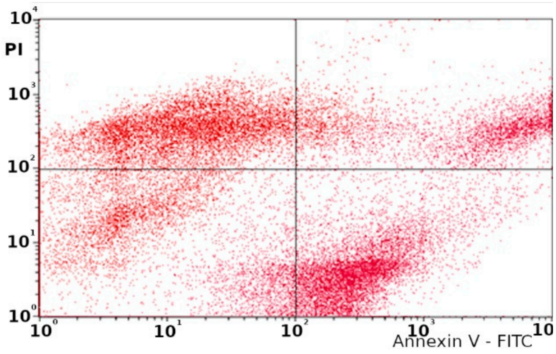

F - L4Apt-5FU (1.5 mg lipids + 500 µg 5-FU/mL)

Figure S5. Analysis of apoptosis based on double stain of the cells with annexin V-FITC and propidium iodide and their corresponding dotplots (down-left quadrant—live cells, top-left quadrant—dead cells, top-right quadrant—apoptotic cells, down-right quadrant—preapoptotic cells).
